# The Distribution and Community’s Perception of Flying Fox, *Pteropus vampyrus* in Limbang, a Transboundary Area in Sarawak

**DOI:** 10.21315/tlsr2022.33.3.11

**Published:** 2022-09-30

**Authors:** Jayasilan Mohd-Azlan, Sally Soo Kaicheen, Lisa Lok

**Affiliations:** Institute of Biodiversity and Environmental Conservation, Universiti Malaysia Sarawak, 94300 Kota Samarahan, Sarawak, Malaysia

**Keywords:** Flying Fox, Chiroptera, Mangroves, Spatial Distribution, Community Perception, Keluang, Chiroptera, Paya Bakau, Taburan Ruang, Persepsi Masyarakat

## Abstract

Flying foxes are threatened throughout their geographic range, and there are large gaps in the understanding of their landscape-scale habitat use. This study identified potential habitats in Limbang, Sarawak and informed potential distribution based on dispersal and interview surveys. Here, biological surveys were combined with interviews of local communities in Limbang Mangrove National Park (LMNP), Sarawak to illustrate distribution and the communities’ perception on the protected flying fox (*Pteropus vampyrus*). Mangrove forest areas were surveyed for for the presence of flying foxes and villagers were interviewed regarding the use by flying foxes of agricultural areas and instances of conflict. Boat and questionnaire surveys were conducted for nine days from 18 to 27 February 2021. The surveys did not record any flying fox roosting sites within the national park and was instead observed to fly from Menunggul Island, Brunei into the national park in the evenings and back to Brunei in the mornings. A total of 27 flying foxes were recorded during the boat survey. Flying foxes were detected from 8/154 survey points and their spatial distribution appeared to be concentrated along Sungai Limpaku Pinang. Most respondents were aware of the species while some have directly observed them in fruit orchards, mangroves, rivers and mixed dipterocarp forests. Eleven perception-based questions were presented, and results showed that locality and income were the most influential parameters exhibiting conservation awareness through Boosted Regression Trees (BRT) analysis. Most respondents believe that flying foxes can uplift the local economy through ecotourism opportunities. However, these findings need to be carefully interpreted as the species has a large home range. Hence, long-term monitoring should be established to generate a larger dataset for stronger analysis to better represent the distribution and occurrence of this species in LMNP.

## Abstract (fr)

Keluang adalah spesies terancam di seluruh kawasan taburan mereka, dan terdapat jurang yang besar dalam pemahaman penggunaan habitat berskala landskap spesies ini. Kajian ini bertujuan untuk mengenal pasti habitat berpotensi untuk keluang di Limbang, Sarawak serta menyelami persepsi masyarakat terhadap spesies ini. Gabungan tinjauan bot dan soal selidik di Limbang dapat menggambarkan taburan dan persepsi masyarakat terhadap keluang di Limbang, Sarawak. Tinjauan terhadap keluang di kawasan hutan bakau di Limbang telah dilakukan dan penduduk kampung tempatan di sekitar Taman Negara Bakau Limbang (TNBL) telah disoal selidik selama sembilan hari dari 18 hingga 27 Februari 2021. Tinjauan tidak merekodkan kawasan sarang keluang di dalam taman negara. Namun, keluang kelihatan terbang dari Pulau Menunggul, Brunei ke taman negara tersebut pada waktu petang dan kembali ke Brunei pada waktu pagi. Sebanyak 27 ekor keluang telah direkodkan melalui tinjauan bot. Keluang dikesan dari 8/154 tempat tinjauan dan taburannya didapati tertumpu di sepanjang Sungai Limpaku Pinang. Kebanyakan responden mengenali spesies tersebut manakala ada yang memerhati secara langsung di kebun buah-buahan, bakau, sungai dan hutan dipterokarp. Sebelas soalan berasaskan persepsi telah dikemukakan dan keputusan menunjukkan lokaliti dan pendapatan merupakan parameter yang paling berpengaruh melalui analisis *Boosted Regression Trees* (BRT). Kebanyakan responden percaya bahawa keluang mampu meningkatkan ekonomi tempatan melalui peluang eko-pelancongan. Walau bagaimanapun, hasil soal selidik ini perlu ditafsirkan dengan teliti kerana spesies ini mempunyai kawasan taburan yang luas. Oleh demikian, pemantauan jangka panjang harus dijalankan bagi menjana set data yang lebih besar untuk analisis yang lebih kukuh untuk mewakili pengedaran dan kejadian spesies ini dengan teliti dalam TNBL.

**Highlights**
- The occurrence of flying fox (*Pteropus vampyrus*) is confirmed in Limbang area via surveys and interviews.
- The surveys did not record any permanent flying fox roosting sites within the national park and was instead observed to fly from Menunggul Island, Brunei into the national park.
- Respondents believed that flying foxes could uplift the local economy through bat-watching ecotourism opportunities that could improve public perception on conservation of this species.

## INTRODUCTION

The large flying fox (*Pteropus vampyrus*) is known to occur throughout Sarawak, but its rarity has been reported since the late 80s (Fujita 1988). In the past, large flying foxes were reported to be common in many areas within Borneo (Payne *et al*. 1985; Phillips & Phillips 2018). However, according to Gumal (2004), all colonies in Sarawak are found to be in remote and inaccessible locations, such as peat swamp forests, mangrove forests and freshwater swamps. The large flying fox prefer to roost in mangrove swamp as this ecosystem shelters them from hunting pressure (Epstein *et al*. 2009). It has been postulated that hunting, habitat loss, decreasing food resources and the foraging patches of the large flying fox in Sarawak may have caused this species to be vulnerable to small changes in their preferred habitats (Kessler *et al*. 2018; Phillips & Phillips 2018). The locations of the colonies in Sarawak are poorly known due to their frequent temporal shift in roost site occupation, as the species is comparatively nomadic, with few permanent camps. Their colonies continuously shift across a large landscape from year to year, making any assessment and monitoring of population sizes and trends challenging. This in turn has resulted in Sarawak’s flying fox to be poorly studied, with most research focusing on zoonotic diseases and ecology instead (e.g., Gumal 2004). As such, relatively little is known about the recent distribution and community perception of this species.

Flying fox is “Protected” species in Sarawak under Section 29(2) of the Sarawak Wild Life Ordinance 1998, in which anyone who commits an offence related to the species (hunts, captures, sells, in possession, etc.), and if found guilty, can face imprisonment for one year and a fine of RM10,000. However, under Section 42(1), the legislation also allows flying fox to be eradicated through lethal methods to protect crops and property. At the global level, this species is listed as Near Threatened (NT) by The International Union for the Conservation of Nature (IUCN) Red List 2020 and reports a decreasing population trend (Bates *et al*. 2008). The large flying fox is also listed in the Convention on International Trade in Endangered Species of Wild Fauna and Flora (CITES) Appendix II. Currently, limited information is available on how this species is being traded internationally.

The colonial tree roosting *Pteropus* species in Malaysia face significant threats such as hunting (Epstein *et al*. 2009; Aziz *et al*. 2019), conflict with fruit growers (Aziz *et al*. 2016; Aziz, Clement, Giam, *et al*. 2017) and large-scale habitat loss due to conversion to monoculture and aquaculture (Mohd Azlan *et al*. 2001; Gumal 2004). Many local communities in Sarawak also believe that the large flying fox is a pest as it feeds on the durian flowers (*Durio zibenthinus*), but a recent study showed that they had mutualistic interactions with durian flowers and serve as the pollinators instead (Aziz, Clement, McConkey, *et al*. 2017). Their ability to cover large areas daily suggests they offer significant ecosystem function as seed dispersers and pollinators compared to other smaller fruit bats (Taylor & Tuttle 2019).

Population surveys are considered to be an essential initial step in determining management and protective needs for a species (Kunz & Jones 2000; Mickleburgh *et al*. 2002) that can provide a basis for judging the success of management programs. Attempts to conserve these declining species from habitat destruction may be hindered by a lack of sufficient information on its status and population trends (Mohd-Azlan *et al*. 2020). Up to date, distributional records of the species are lacking, and ecological studies have been neglected and have not been brought up to date since the investigations by Gumal in 2001 and 2004 (Gumal 2001; 2004) in Sarawak. Understanding how wide-ranging animals utilise landscapes that overlap with human use is essential to understand patterns of human-wildlife conflict, disease transmission, and to design mitigation strategies. Therefore, this study attempts to update the current occurrence records relevant to the species management in this region. Little is known about the perception, knowledge and level of awareness about flying fox of the local communities in the Limbang area, as no prior studies have been conducted on these aspects. As community-based wildlife surveys are known to be an effective tool to help elucidate the distribution of wildlife species (Fitzgibbon & Jones 2006), we have included this approach to investigate the flying fox occurrences and the perception of the local communities.

## MATERIALS AND METHODS

### Data Collection

#### Field data collection

**Figure 1:**
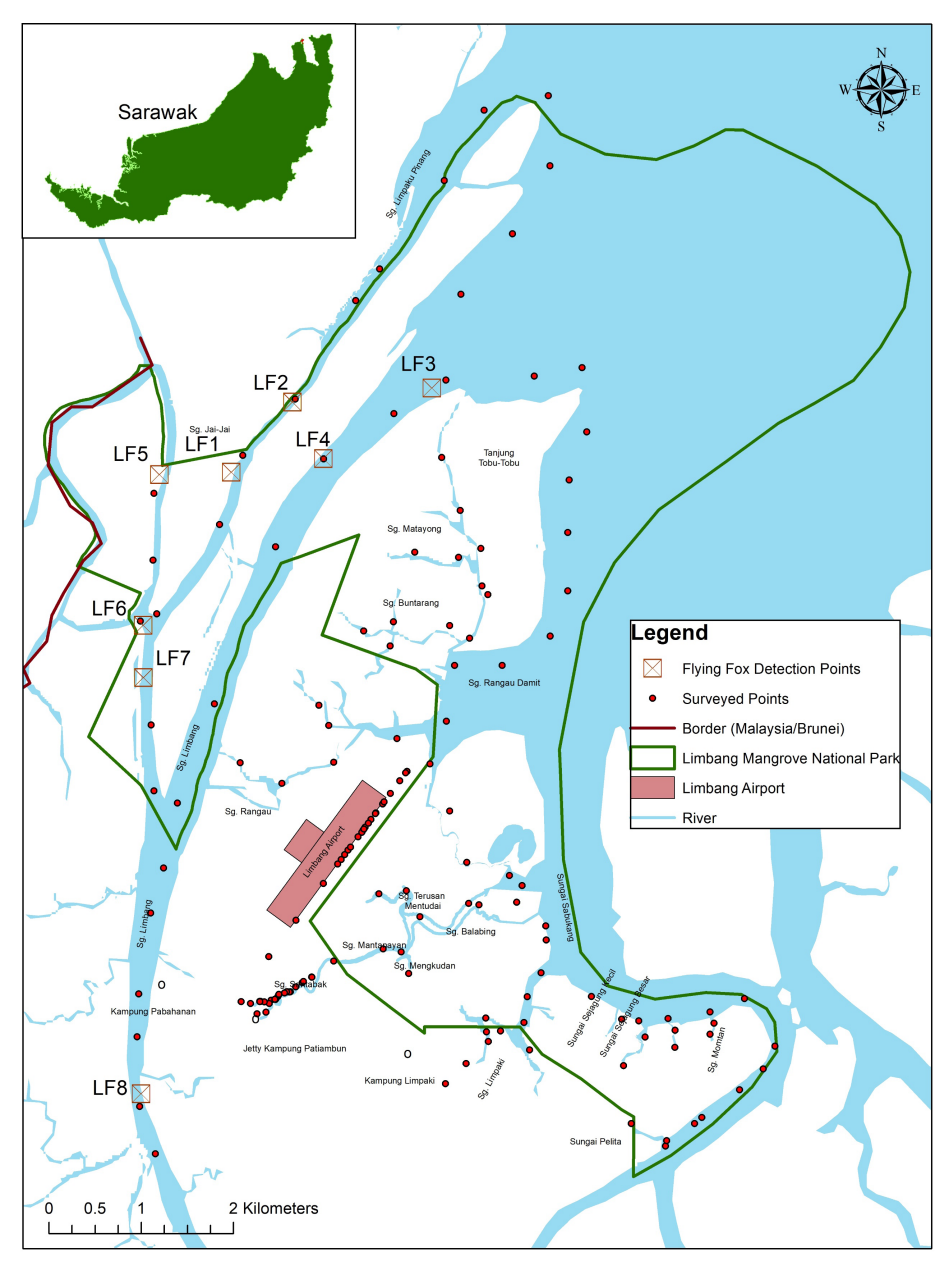
The locations of the surveyed points in Limbang area including Limbang Mangrove National Park (LMNP). Eight locations (LF1– LF8) where the large flying fox (*Pteropus vampyrus*) were recorded with at least one individual sighting from 18 to 27 February 2021 in the early mornings and evenings *Source*: SFC, ArcMap.

The survey was conducted along the roads and rivers to reach the mangroves. Land surveys covered areas nearby Limbang Airport, Jalan Rangau and Kampung Patiambun while boats were used in the mangrove to gain access to other potential sites. The boat surveys were carried out along Batang Limbang, Sungai Limpaku Pinang up to Kuala Limbang, Sungai Rangau Damit, Sungai Buntarang, Sungai Matayong, Sungai Sentabak (Second Order of Sungai Sentabak – Sungai Mengkudan, Sungai Mantanayan, Sungai Ungsang, Sungai Uching, Sungai Sembilang, Sungai Terusan Mentudai, Sungai Balabing), Sungai Limpaki, Sungai Pandaruan, (Second Order – Sungai Rangau, Sungai Kibi and Sungai Temburung), Sungai Sejagung Kecil, Sungai Sejagung Besar, Sungai Momtan and Sungai Pelita (Fig. 1). These surveys were conducted daily from 18 to 27 February 2021, during the early mornings and evenings. Additionally, a drone was deployed to scout for potential roosting sites.

A dispersal count of individuals leaving and returning to their roosts was conducted according to methods by Mohd-Azlan *et al*. (2001). Solitary bats were counted as they emerged from the roosting site to feed before darkness. The observation was difficult in these instances as bats departed from several locations within the survey area and dispersed into several directions. Dispersal counts were typically conducted between 0550 h–0830 h and 1800 h–1930 h. Several observation areas were selected based on previous sightings so that most departing bats could be silhouetted against the sky. The numbers of bats were tallied with a hand counter by two observers until 1930 h as darkness prevented further observation and records. Once the emerging bat was observed, the location’s coordinate was taken using a GPS (GPSMAP^®^ 64s) and recorded in the datasheets.

#### Questionnaire survey

A questionnaire survey consisting of open-ended and fixed response questions was designed to obtain data on the (1) local community socio-demographics; (2) flying fox sightings; (3) consumption and hunting patterns of flying fox; and (4) local community perceptions on flying foxes (Appendix A). The answers were organised using 5-point Likert scale with the combination of positive and negative statements. In order to reduce physical contact during the pandemic, the survey was administered through a printed questionnaire and a softcopy version in Microsoft Forms in the Malay language. Technicians were trained in interviewing protocols and assessment procedures by the primary investigator prior to the study. As part of this process, technicians viewed and participated in several mock interviews with the primary investigator and one another, serving as both interviewee and interviewer. A pilot survey was first conducted on 15 individuals comprising of the public and students from Universiti Malaysia Sarawak (UNIMAS) from Kota Samarahan. Questionnaires were then distributed to local communities opportunistically, particularly to those residing near the mangroves (Kampung Pabahanan, Kampung Patiambun, Kampung Limpaki), Limbang sub-urbans (Kampung Seberang Kedai, Kampung Pahlawan, Kampung Ukong, Kampung Sembiling, Taman Bunga Raya, Kampung Sibukang, Kg Tegarai), staff members from Sarawak Forestry Corporation Limbang and Sarawak Regional Marine Department. The questionnaire survey was conducted from 16 to 28 February 2021. Variables used in the perception and awareness survey is included in the Appendix B.

### Data Analysis

#### Software

The collected data were analysed and plotted in the software R version 4.0.3 with various packages, (dismo, gbm) while the spatial distribution illustrated with ArcMap version 10.2.0.

#### Kernel Density Estimation (KDE)

The spatial distributions and occurrence density of flying fox were visualised using the “Kernel Density” tool in ArcMap 10.2 (Esri 2021). Kernel density estimates the magnitude per unit area from presence-only data, either point or polyline, which fits the occurrence data from the same study site into a smooth tapered surface. This study used the point data, the locations where the flying foxes were detected during the survey. The KDE visualised the frequency data robustly compared to other methods (Spencer *et al*. 2017), where the density at a new location (x, y) is predicted with the following formula:


$$\begin{array}{c} Kernel\;Density=\frac{1}{{\left(radius\right)}^{2}}\sum_{i=1}^{n}\left[\frac{3}{\pi }\cdot {pop}_{i}{\left(1-{\left(\frac{{dist}_{i}}{radius}\right)}^{2}\right)}^{2}\right]\\ \text{For }{\text{dist}}_{\text{i}}<\text{radius} \end{array}$$

where *i* = 1, …, *n* are the sighting locations. The locations were only included if they were within the radius distance of the (x, y) location; *pop_i_* is the population field value of point *i*, yet is an optional parameter; while *dist_i_* represents the distance between points *i* and the (x, y) location. Our study used a cell size of (0.0001133639, 0.0001133639) and the number of bands = 1. A smaller value of the radius parameter is suggested to produce a raster with more details. The radius was entered manually when the mean of the population field was much smaller than 1. Otherwise, the default radius shall be sufficient (Esri 2021).

#### Boosted Regression Trees (BRT) for ecological modelling

Local communities were asked on their encounters with flying foxes. The BRT analysis with a predictive linear model was carried out to relate the respondents’ demographics with their answers based on the 5-point Likert scale to the flying fox perception survey, using demographics as predictors and questions as variables (Appendix B). BRT is an analytical method that evaluates the respective strengths of predictor variables that would affect the outcome of an analysis. Identification of the strongest predictor variable was conducted by combining the strengths of two algorithms: regression trees and boosting. Regression tree models relate a response to its predictors by recursive binary splits while boosting allows the combination of many simple models to obtain an enhanced prediction. The responses from the respondents were structured and analysed with the R package “dismo” and “gbm”. This is a modified model and function in “gbm” package which allows for the application of ecological data to be more efficient and enhances interpretation at the same time (Elith & Leathwick 2017).

## RESULTS

A total of 154 survey points were established in LMNP. However, the survey did not reveal any roosting sites within the boundary of the national park. Flying foxes were seen leaving mix dipterocarp forests from Menunggul Island, Brunei (Appendix C). The local community claims that there were no permanent roosts of the flying fox within the national park boundary but reported a temporary roost near Tanjung Tobu Tobu in the late 2020s. However, flying foxes have been observed by the observers to fly across the mangroves during the late evenings and mornings. In the evenings, the flying foxes were seen flying at the heights of approximately 50 m–80 m above the ground from Brunei (Menunggul Island) towards Limbang mangroves. While in the early morning, flying foxes were spotted returning towards the same direction of departure. A total of 27 individuals flying foxes were recorded during their flight throughout the 14 sampling sessions (dusk and dawn surveys). The flying foxes were only recorded 21.4% (*n* = 3) of the sampling sessions, ranging from a single to 11 individuals.

### Spatial Distribution of Flying Fox

Flying foxes were only detected in 5.2% of the total surveyed area throughout the 14-sampling sessions (dusk and dawn surveys), indicating that they are transient within LMNP. Flying foxes were detected at eight locations throughout the survey. The first location in which two individuals of flying foxes were detected was recorded as LF1. The pair was observed flying across the river at 0638 h nearby the Sungai Limpaku Pinang. The flying foxes were around 50 m away from the shoreline with an approximately flying height of 100 m in the air. Another group of flying foxes (11 individuals) were spotted immediately after at 0648 h and its location was marked as LF2. The group was flying towards the international boundary of Brunei and were approximately 30 m away from the river. Another two individuals were recorded at two locations, respectively. The sites were marked as LF3 and LF4 and observation was for 0827 h and 0845 h. These detection points were located at Sungai Limbang, whereby LF3 was close to Tanjung Tobu Tobu. Flying foxes at LF3 was observed flying at heights of 60 m while the flying foxes at LF4 was relatively lower, about 20 m in the air.

Subsequently, three groups of flying foxes were spotted along the Sungai Limpaku Pinang. The first group (LF5) consisted of two flying foxes, which were seen flying across the river at around 1835 h at the mangrove areas. The flying foxes were observed to be flying around heights of 100 m and were moving from Brunei towards the Limbang area. Another four flying foxes were observed at 1853 h at the location marked as LF6 and were flying in the same direction as the LF5 group. This group had a lower flight height of approximately 80 m. Detection point LF7 recorded five individuals of flying foxes at 1901 h at a flying height of 80 m and in the direction of Brunei towards Limbang. These detection points were all situated at the mangrove forests. At LF8, only one individual was recorded along Sungai Limbang at 1850 h flying from the direction of Brunei towards Limbang and observed to be much closer to the Limbang town area than the other detection points.

The detection points of flying foxes in Limbang were also measured with the distance from the Limbang Airport. The Limbang Airport is located approximately 500 m from the nearest boundary of LMNP. LF3 was the furthest detection point from the airport with approximately 4.3 km away. This detection point (LF3) however, recorded only one flying fox. In contrast, the second furthest detection point from Limbang Airport (LF2) recorded the highest numbers of individuals (*n* = 11). LF2 was around 4.15 km away from the Limbang Airport.

### Perception and Awareness of the Local Community on the Distribution and Conservation of Flying Fox

**Figure 2:**
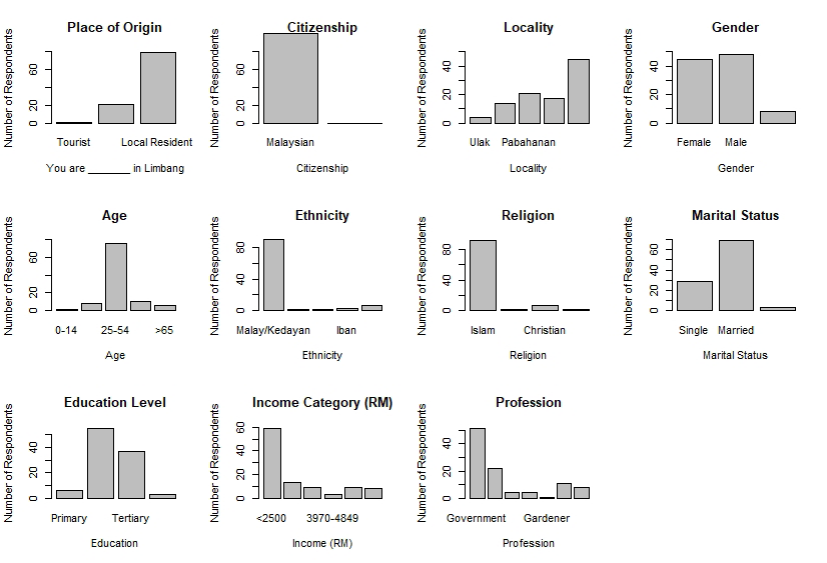
Socio-demographics of 101 respondents showing their place of origin, citizenship, village, gender, age, ethnicity, religion, marital status, education level, income category (RM) and their profession in Limbang area.

Throughout the sampling period, a total of 101 responses were collected from the local communities and government agencies. Out of the 101 respondents, 79 were residents (78.2%), 21 (20.8%) were either working or spouses of those working in Limbang, and one tourist (1.0%) (Fig. 2). All the respondents were Malaysian citizens including the local tourist.

The majority of the respondents were from Kampung Pabahanan (20.8%), Kampung Patiambun (16.8%), Kampung Limpaki (13.9%), Kampung Ulak (4.0%), while 44.6% were from other areas in Limbang. The respondents consisted of 47.5% males, 44.6% females while the remaining 7.9% chose not to disclose their gender. In terms of age group, most of the respondents (75.3%) were between 25–54 years old and were in the prime working-age group, while 10 respondents (9.9%) were between 55–64 years old (mature working age), 8 respondents (7.9%) were between 15–24 years old (early working age), six respondents (5.9%) were elderly (> 65 years old) and one child (1.0%) was between 10–14 years old. The highest frequency for respondents’ ethnicity was Malay/Kadayan Muslim (89.1%; *n* = 90), others (5.0%; *n* = 5), followed by Iban (3.0%; *n* = 3), Bisaya (1.0%; *n* = 1), and Chinese (1.0%; *n* = 1). Six respondents did not include their ethnicities during the survey. Approximately 68.3% of the respondents were married, 28.7% were single, and three (3.0%) did not disclose their marital status (Fig. 4). Majority of the respondents (54.5%) had a secondary school educational attainment, followed by tertiary attainment (36.6%), primary school (5.9%) and three respondents (3.0%) did not receive any formal education.

With regards to the income level of the respondents, most (59.4%) received a salary below RM2,500 (B1 category in B40 group), 12.9% between RM2,501–RM3,169 (B2 category in B40 group), 8.9% between RM3, 170–RM3, 969 (B3 category in B40 group), and 3.0% between RM3, 970–RM4, 849 (B4 category in B40). Another 8.9% of respondents were from the M1 category of the M40 group with a salary range of RM4, 850–RM5, 879. Yet 7.9% of respondents did not reveal their salary ranges (Fig. 2). The majority of the respondents (50.5%) were working as government servants, 21.8% were working in private companies, 4.0% were fishermen, and 4.0% were students. Approximately 10.9% of the respondents were not working, one respondent (1.0%) was a gardener/farmer, and eight respondents (7.9%) had various other professions (Fig. 2).

### Sightings of Flying Foxes in Limbang

**Figure 3:**
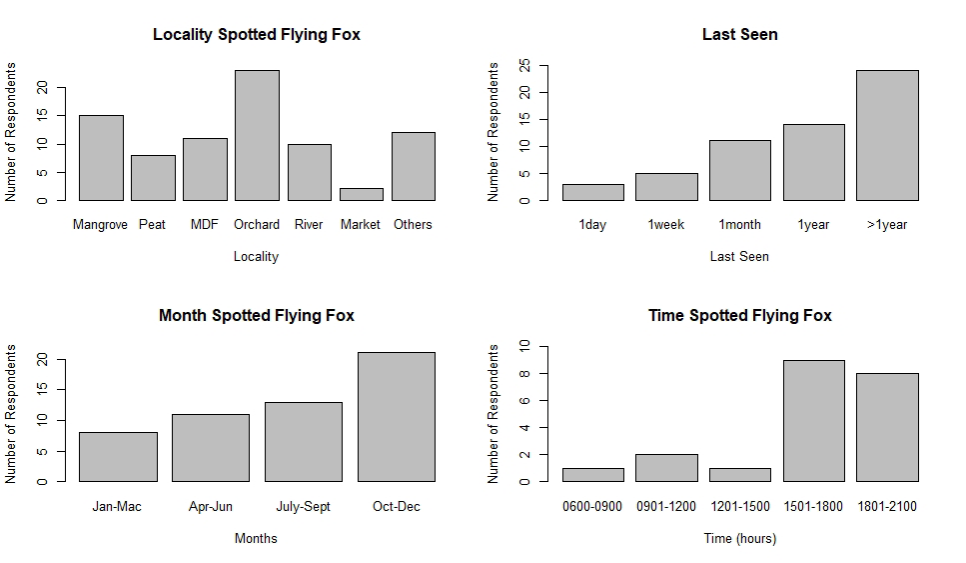
Data from 58 respondents who have seen a flying fox in Limbang categorised according to habitats, last seen, month and time of observation.

Out of the total 101 respondents, 58 (57.4%) reportedly saw a flying fox in the Limbang area (Fig. 3). The probability of observing a flying fox appears to be influenced by residency, in which 80 respondents (79%) were residents in Limbang. Most records of flying fox observations were from fruit orchards (39.7%, 23/58 respondents), followed by mangroves (15/58; 25.9%) while 12 respondents (20.7%) had observed flying foxes from areas that were not stated in the survey options. Another 11 respondents claimed to have seen flying foxes at mixed dipterocarp forest (19.0%), 10 respondents observed the species flying across rivers (17.2%) and two respondents claimed to have seen it sold in the markets but did not disclose the location of which market/tamu.

**Figure 4:**
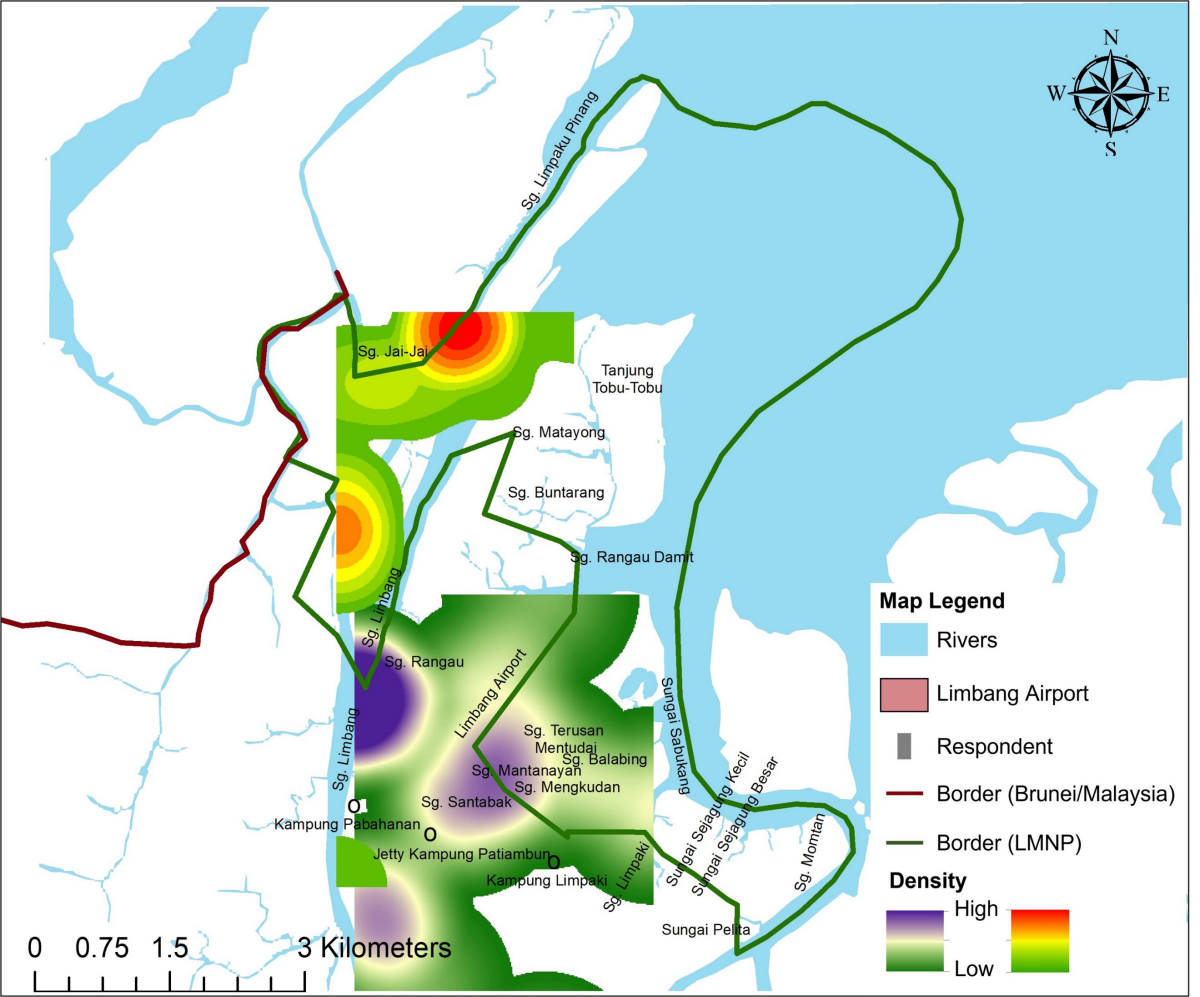
The combination of heat map based on the detection points of flying fox in LMNP through observation and questionnaire surveys, the location where flying foxes were spotted and where respondents claimed they had seen a flying fox. The colour indicates the intensity of occurrence ranging from green (low) to red (high) (based on observations), green (low) to purple (high) (based on questionnaire surveys). The green line indicates the boundary of LMNP, while the red line indicates Brunei and Malaysia’s border. *Source*: SFC, ArcMap

However, most of the sightings were from more than a year ago, during the flowering and fruiting season (October and December 2019) of various fruits such as rambutan (*Nephelium lappaceum*) and durian (*Durio* spp). Flying foxes were also frequently spotted in the late evening, during dusk and throughout the night from 1501 h–2100 h (Fig. 4). The localities where respondents saw flying foxes were mainly concentrated at Sungai Limbang and Sungai Santabak. The respondents also mentioned that they observed flying foxes flying across their villages at Kampung Pabahaman, Kampung Patiambun and nearby the Limbang Airport (Fig. 4).

Only a few respondents (6.0%) claimed to have consumed flying foxes in this area. As such, the BRT analysis could not reveal any significant relationships between the social-demographics and their dependency on flying foxes. Respondents also declared that they obtained the meat of flying foxes through their friends; either found dead or through hunting. Information on hunting was also gathered to investigate hunting pressure adjacent to the residential area. Out of the six respondents who consumed flying fox meat, only two individuals declared that they have hunted flying foxes while one respondent claimed he hunted for flying foxes but has never consumed one. These three respondents (two male one female) were all local residents. One of them was between 25–54 years old, while the other two were aged between 55–64 years old.

### Perception and Boosted Regression Tree (BRT) Interpretations

The last 11 perception questions in the survey were on the effectiveness of approaches related to flying fox’s conservation efforts. The BRT model output showed that respondents’ income (perception item 8) and locality (perception item 2) were the two most impactful predictor variables in the perception survey statement. While age, place of origin, religion, gender made up the minor variables influencing the respondents’ answer patterns (Table 1).

Three out of the 11 perceptions received “unsure” as the most frequent answer. Firstly, a total of 64 respondents (63.4%) were uncertain whether consuming flying fox meat is good for health. The BRT output highlighted income (77.1%; lower income) as the most impactful predictor for this perception statement, followed by education (13.7%), where they resided (locality; 4.6%), profession at 4.1%, residency (local/non-local; 0.3%), marital status (single/married; 0.2%), ethnicity (0.1%). While gender, age, and religion did not have any effects on the perception’s answer pattern (Table 1).

Approximately 60.4% of the respondents (61 out of 101 respondents) were also unsure of the flying fox’s role in seed dispersal (perception 2). This perception was largely influenced by the respondents’ profession type (54.1%; mostly are government servants) while age group (20.8%) was the second largest predictor in the BRT model. Income level was ranked as the third predictor (19.8%), followed by where they reside (locality; 4.1%), marital status (0.7%) and education (0.5%). Other demographics such as gender, place of origin (originally from Limbang or from elsewhere in Malaysia), ethnicity, and religion did not seemingly influence the answer patterns (Table 1).

Subsequently, 43 of the respondents (42.6%) were unsure whether occasionally consuming flying fox meat was acceptable or not (perception 6). The answers were mainly associated with the respondents’ income (59.5%, lower income), followed by locality where they reside/lived (12.3%), profession (10.4%), age (8.9%) and place of origin (5.7%). Other variables which influenced the answers were ethnicity (1.3%), education (1.2%), gender (0.4%) and marital status (0.2%). Religion was found to have no effects (Table 1).

For perception 3 (the role of flying foxes in promoting tourism), respondents’ income (58.2%, higher income) was the strongest predictor. Nested under, 57.4% of the respondents had a positive outlook on the flying fox’s role in promoting tourism. The locality where they resided/lived (from Kampung Ulak, Limpaki, Pabahanan and Patiambun) was the second most influential predictor variable (21.9%), followed by place of origin (local Limbang/non-local; 12.6%), profession (3.6%), ethnicity (1.9%) and age (1.6%). The other predictor variables (gender, status, education, and religion) had no effects on the answering patterns for this perception item (Table 1).

Respondents also believe that constructing a jetty in the mangrove forest would enhance the flying foxes-based tourism (52.5%). The most vital variable for the respondents’ pattern in reacting to the statement was highly influenced by their income (higher income), with a relative inference of 45.4%, followed by where they lived (locality; 27.2%, mostly from Kampung Ulak and Limpaki), education (12.3%), age (9.9%), profession (3.3%), ethnicity (1.8%) while gender, place of origin and marital status were 0.1%, respectively. Religion remains to be a non-influencing factor (Table 1).

Additionally, 54.5% of respondents believed that flying fox based ecotourism activities could potentially benefit the local economy. This perception item was highly influenced by respondents with higher income (57.1%). Respondents were aware of the potential of this industry in enhancing their living standards. After income, variables that contributed to the answer patterns were; where the respondent lived (locality; 17.8%), age (10.4%), education (5.7%), profession (4.2%), ethnicity (2.5%), marital status (1.8%) and gender (0.4%). Two other predictor variables (place of origin and religion) did not seem to influence the choices (Table 1).

Furthermore, 41.6% of the respondents agreed and 25.7% strongly agreed that hunting and selling flying fox meat could damage the species’ populations in the long term. Income was identified as the variable with the highest impact (55.0%; higher income) for this perception item and was followed by the locality where respondents reside (40.0%), profession (2.1%), marital status (1.1%) and gender (0.7%). Ethnicity and age contributed equal values of relative inference (0.4%) while education was 0.3%, place of origin was 0.1% and religion was found to have no inferring effects on the perception (Table 1).

Majority of the respondents (69.3%) also agreed that deforestation could lead to greater negative impacts on the flying fox populations compared to hunting. The income variable was determined to have the highest inference (49.2%; higher income) on this perception, followed by profession (45.4%) (Table 1).

Most respondents had positive feedbacks on conducting awareness programs in schools to increase the conservation efforts for flying fox (62.4%). The income predictor was most significant (60.4%; higher income) for this perception followed by profession type of the respondents (19.8%) and locality (15.3%), as shown in Table 1.

Lastly, locality (where respondents lived) was the highest contributing factor in influencing the respondents’ perceptions on the necessity of wildlife law in Sarawak to protect flying foxes (67.3%; mostly from Kampung Ulak, Limpaki, Pabahanan, Patiambun). This predictor (locality where they lived) also affected the respondents’ choices on their role in preserving flying fox (63.4%) at the locality level. Profession was the second-best predictor (19.60%), followed by age (14.7%).

In addition to the existing Sarawak Wild Life Law the local communities also acknowledged their role in protecting the flying foxes. Where the respondent lived was the largest contributing predictor (69.6%; mostly from Kampung Ulak and Limpaki) followed by age (13.1%), income (9.4%) and profession (7.1%). Place of origin, gender, ethnicity, and religion demonstrated no effects on the answering pattern. In general, the results suggest that most respondents in Limbang believe that conserving the flying fox is essential for generating a sustainable income through eco-tourism (Table 1).

## DISCUSSION

The population status of flying foxes in Sarawak is relatively unknown due to the lack of consistent and exhaustive population studies across the state. The last flying fox population survey conducted in Sarawak was approximately 20 years ago by Gumal (2001), between 1997 and 2000 discovering that there were only maternity colonies of flying foxes at Patok Island, Loagan Bunut, Sarang, Limbang and Sedilu. However, their study did not reveal any evidence on flying foxes’ permanent roosts in LMNP even though mangrove and peat swamp forests are their common roosting sites (Struebig *et al*. 2007). Current estimates on abundance and population sizes in LMNP cannot be accurately assessed due to incomplete or lacking information on the roost locations in Brunei.

Satellite tracking of flying fox in Peninsular Malaysia has shown that this species can forage over distances of up to 87.5 km in one night (Epstein *et al*. 2009). Based on research in Sarawak (Gumal 2001), along with anecdotal reports from local communities (Mohd-Azlan unpublished data), flying foxes are attracted to durian flowers and may have a strong relationship as an important long-distance pollinator for this tree species (Struebig *et al*. 2007; Aziz, 2019). Additionally, *Sonneratia* sp., a mangrove species, has also been documented in the flying foxes’ diet (Gumal 2001). During our study, groups of flying foxes were observed to be flying from the direction of Brunei towards Limbang during the evenings, and in the opposite direction in the mornings. Hence, it is assumed that the flying foxes in this area are most likely to utilise LMNP and other forested regions within Limbang as foraging sites. This also supports the fact that this species may play an essential role as pollinators and seed dispersal agents across various ecosystems (Mix dipterocarp forest and mangrove forest) and landscape types (durian orchards) in the Limbang area.

This study’s findings clearly demonstrated that most of the survey respondents were aware of flying foxes in Limbang, however the species presence was presumed to be uncommon in the area. Despite more than half of the respondents claimed to have previously observed a flying fox in Limbang, they did not mention or clearly remember the exact location. Around one third of the respondents who have seen flying foxes specified the locality in which the species was observed, and these sightings seemed to be rather opportunistic. Some mentioned that the flying foxes were mostly flying across orchards, rivers, villages and even near the airport area.

This study also raises concern on the flying fox’s population, as they may be facing a decline throughout the years attributed to urban development since most respondents expressed sightings to be more than a year ago. Based on recent observations and questionnaire surveys, flying fox populations were frequently observed near Sungai Limpaku Pinang and Sungai Limbang (Appendix D). Their occurrences in these areas further establish the notion that flying foxes utilise LMNP as an important foraging spot.

The majority of the respondents recognised flying foxes as an iconic species in promoting the eco-tourism industry in Limbang. This finding is consistent with another study by Mohd-Azlan *et al*. (2022) in western Sarawak. Sustainably managed and regulated bat tourism can potentially contribute to bat conservation (Aziz, Clement, McConkey, *et al*. 2017; Tanalgo & Huges 2021). The local community, especially near Kampung Patiambun also agreed that constructing a jetty could further enhance the sector and thereby would enhance the economy of the local community. The respondents, however, were unsure of the benefits of consuming flying fox meat, and indirectly influenced their decision on whether they occasionally consume flying fox meat is appropriate or not. Yet, they supported the awareness programme to be held in school on flying fox’s conservation.

Most of the respondents were not aware of the ecological functions that the flying fox provide in the ecosystem (e.g., seed dispersal agent) and this has perhaps influenced their perception to regard this species as a pest, as flying foxes were always spotted in their orchards. Increased awareness and knowledge on flying foxes were found to contribute to the conservation of the species by local communities (Jaunky *et al*. 2021). This occurrence is common in Southeast Asia whereby the flying foxes were observed to “fruit-crop raid”, however the issue remains poorly understood (Aziz *et al*. 2016). Nevertheless, this perception did not result in any human-wildlife conflicts as respondents were mindful of the governmental protection status of the species under the Sarawak Wild Life Ordinance 1998. This could be because most of the respondents are government servants. The respondents were also aware that deforestation and hunting has reduced the flying fox local population size.

Most of the perception survey responses showed that higher income was the strongest predictor, suggesting that the respondent’s income level significantly influenced their perceptions on flying foxes. Another significant predictor was the respondents’ ethnicity; the Malay/Kadayan Muslim. Ethnicity also appears as an influential predictor for a study on hornbills and cultural beliefs in Sarawak (Phillovenny & Mohd-Azlan 2021). This suggest that flying fox conservation can be incorporated into customary law at the grassroots level in Limbang. Limbang has a total population of 48,186 residents in which the Malay/Kadayan were the largest group (31.3%), followed by Iban (30.4%), Chinese (16.0%), Bisaya (13.1%), Lun Bawang/Kelabit (5.4%) and other ethnicities (3.7%). Many respondents in this survey consisted of Malay/Kadayan Muslims. Hence, information on flying foxes hunting around LMNP was limited since the Malays do not participate in the traditional hunting activities (Bennett 2000; Struebig *et al*. 2007; Yi & Mohd-Azlan 2020).

Public support is considered as an important factor in the protection of species of conservation importance (Hatch *et al*. 2002). Our survey has provided some novel information on the relationships between local communities and flying foxes near the Limbang mangroves. At the same time, this exercise also yielded details to understand the current situation better, guiding appropriate conservation strategies. For example, while conducting this survey, we found that flying fox meat was not seen in markets (including tamu), which partially indicated the authorities’ level of awareness and enforcement activities. Additionally, only six out of 101 respondents (5.9%) have declared consuming flying fox, while only three respondents claimed to have hunted. These numbers were much lower than expected, suggesting that flying fox meat is not a significant source of protein for the Limbang’s residents. Additionally, cultural differences between respondents and fear of being prosecuted, it is possible that some people did not want to declare such consumption. However, respondents have also claimed that outsiders (not Limbang’s residents) have attempted to hunt this species a few years ago near Kampung Patiambun. The local villagers then stopped this activity, highlighting their level of awareness on protecting wildlife.

However, it is essential to remain attentive in monitoring and preventing mangrove deterioration around the protected area, mainly to avoid losing important foraging sites for flying foxes. The questionnaire survey recorded that flying foxes were spotted flying across Sungai Rangau, Limpaki and Kampung Patiambun, whereby Sungai Rangau and Kampung Patiambun are outside the protected area (Appendix E). Hence, any significant clearance of mangroves outside the protected area should be considered a direct threat to the species.

Flying foxes are also seen as a potential threat to aircrafts and have been regarded as a safety issue in the tropics (Parsons *et al*. 2008). Collisions can sometimes be damaging and hazardous due to their large body mass, congregation patterns and the species ability to fly near aircraft heights. To date, there have been no flying fox collision cases with aircrafts in the Limbang area as there were no early and late evening flights in Limbang that are consistent with the dispersal and return flight schedules of this species.

The development of effective conservation plan for flying fox in LMNP will depend on the determination of realistic and achievable targets, an appreciation of the conservation and environmental contexts in the area, and reliable data on the species’ distributions and ecology. Considering this, several recommendations were prescribed for consideration:

1. Continue to monitor the occurrence of flying foxes in LMNP. If a temporary roost is located, they need to be recorded and characterised, including human accessibility and distance to human presence, to understand roost locations better and protect important roost sites within the protected area.
2. Conduct GPS tracking studies of individuals to accurately identify and map key foraging areas and vegetation communities utilised by flying foxes in LMNP.
3. Conduct annual and/or monthly exit counts of evening fly-outs, as close in time as experienced or trained observers/biologists using standardised, readily repeatable methods to understand their occurrence in the Limbang area.
4. Promote understanding and awareness of flying foxes through regular dissemination of information through community engagement, posters, electronic media, and organising or participating in public events, highlighting the essential roles of flying foxes and other fruit bats as pollinators and seed dispersers.
5. Educate the local community on potential disease risk (zoonotic diseases) and emerging infection diseases that may stem from flying foxes to deter human contact with the species and its consumption.
6. Involve the local community in citizen science by involving them in reporting initiatives and establishing a community database. Data such as flight path identification and roost locations can be contributed by the local community to generate larger data to improve analysis and generate the local interest in research.

## Figures and Tables

**Table 1:** List of answers, Boosted Regression Tree (BRT) models that relate the respondents’ demographics on perceptions. The respondents’ demographics used as the variance of the BRT model. In contrast, the relative inference values represent the strength of the predictor variables, the demographics.

Perception 1: Consuming flying fox meat is good for health.										
MFA	BRT Model (%)									
Unsure	Income	Education	Locality	Profession	Residency	Status	Ethnicity	Gender	Age	Religion
	77.10	13.70	4.60	4.10	0.30	0.20	0.10	0.00	0.00	0.00
**Perception 2: Flying fox plays an important role in seed dispersal**.										
Unsure	Profession	Age	Income	Locality	Status	Education	Gender	Residency	Ethnicity	Religion
	54.10	20.80	19.80	4.10	0.70	0.50	0.00	0.00	0.00	0.00
**Perception 3: Flying fox plays an important role in promoting tourism**.										
Agree/strongly agree	Income	Locality	Residency	Profession	Ethnicity	Age	Gender	Status	Education	Religion
	58.20	21.90	12.60	3.60	1.90	1.60	0.00	0.00	0.00	0.00
**Perception 4: The tourism industry (flying fox) can be enhanced by building jetty in the mangrove forest**.										
Agree/strongly agree	Income	Locality	Education	Age	Profession	Ethnicity	Gender	Residency	Status	Religion
	45.40	27.20	12.30	9.90	3.30	1.80	0.10	0.10	0.10	0.00
**Perception 5: Flying fox tourism activities can increase the local economy**.										
Agree/strongly agree	Income	Locality	Age	Education	Profession	Ethnicity	Status	Gender	Residency	Religion
	57.10	17.80	10.40	5.70	4.20	2.50	1.80	0.40	0.00	0.00
**Perception 6: Occasionally consuming flying fox meat is fine**.										
Unsure	Income	Locality	Profession	Age	Residency	Ethnicity	Education	Gender	Status	Religion
	59.50	12.30	10.40	8.90	5.70	1.30	1.20	0.40	0.20	0.00
**Perception 7: The hunting and selling of flying fox can damage flying fox populations in the long term**.										
Agree/Strongly Agree	Income	Locality	Profession	Status	Gender	Ethnicity	Age	Education	Residency	Religion
	55.00	40.00	2.10	1.10	0.70	0.40	0.40	0.30	0.10	0.00
**Perception 8: Deforestation causes a more negative impact on the flying fox population compared to hunting activity**.										
Agree/Strongly Agree	Income	Profession	Ethnicity	Locality	Age	Residency	Gender	Status	Education	Religion
	49.20	45.40	1.90	1.10	1.00	0.80	0.30	0.20	0.10	0.00
**Perception 9: Wildlife law is necessary for Sarawak to protect flying fox**.										
Agree/Strongly Agree	Locality	Profession	Age	Education	Income	Gender	Status	Ethnicity	Residency	Religion
	45.50	19.60	14.70	8.40	8.20	1.70	1.00	0.80	0.20	0.00
**Perception 10: Besides the Sarawak Wild Life Law, flying fox also needed to be protected at the locality level**.										
Agree/Strongly Agree	Locality	Age	Income	Profession	Education	Status	Residency	Gender	Ethnicity	Religion
	69.60	13.10	9.40	7.10	0.60	0.10	0.00	0.00	0.00	0.00
**Perception 11: Awareness program in schools will help to increase the effort to conserve the flying fox**.										
Agree/Strongly Agree	Income	Profession	Locality	Age	Residency	Gender	Ethnicity	Religion	Status	Education
	60.40	19.80	15.30	4.40	0.00	0.00	0.00	0.00	0.00	0.00

*Notes*: MFA – Most Frequent Answer

## APPENDICES

### Appendix A

The questionnaire survey about flying fox consisting of open-ended and fixed response questions in Malay.

### Appendix B

Variables used in the perception and awareness of the local community on the distribution and conservation of flying fox in Limbang.

**Table:**

Category	Variable	Definition		Data Type
Socio-demographics	Place of origin	Local, Working/spouse is working, Tourist		Categorical
	Citizenship	Malaysian, Non-Malaysian		Categorical
	Locality	Which village are you staying?		Categorical
	Gender	Male, Female		Categorical
	Age	10–14 years old, 15–24 years old, 25–54 years old, 55–64 years old, >65 years old		Categorical
	Ethnicity	Iban, Melayu/Kadayan, Chinese, Bisaya, Lun Bawang/Kelabit, Penan, Tabun, Others		Categorical
	Religion	Christian, Islam, Buddhism, Atheist, Taoism, Bahai, Others		Categorical
	Marital status	Single, Married		Categorical
	Education	Primary school, Secondary school, Tertiary school, Never been to school		Categorical
	Income	<RM2500, RM2501–3169, RM3170–3969, RM3970–4849, >RM4850		Categorical
	Profession	Government, Private, NGO, Fisherman, Student, Not working, Gardener, Others		Categorical

Flying fox sightings	Have you ever seen a flying fox?	Yes, No		Binary
			
	If yes, where did you see it? (Can choose more than one)	**Habitat**	**Name of the area**	Categorical
			
		Mangrove		
			
		Peat Swamp		
			
		Mixed Dipterocarp		
			
		Orchard/Open Space		
			
		River		
			
		Market		
			
		Others (………….)		
			
	If you had ever seen a flying fox, when was the last time?	One day before, one week before, one month before, one year before, > a year		Categorical
	If you had ever seen a flying fox, which month you saw?	January – March, April – June, July – September, October – December		Categorical
	What time have you ever seen a flying fox?	0600 – 0900 hrs, 0901 – 1200 hrs, 1201 – 1500 hrs, 1501 – 1800 hrs, 1801 – 2100 hrs, 2101 – 0000 hrs, 0001 – 0300 hrs, 0301 – 0600 hrs		Categorical
	As you know, is there any villager in your village who is still hunting flying fox? If yes, how many villagers?	None, 1 – 3 people, 3 – 6 people, 6 – 9 people, 9 – 12 people, >12 people		Categorical
	If yes, when was the last time they hunt?	< a year ago, 1 – 5 years, > 5 years ago		Categorical

**Consumption and hunting patterns of flying fox**	Have you ever eaten flying fox meat?	Yes, No		Binary
	If yes, where did you get the supply?	Market, Hunt, Given by a friend, Found dead		Categorical
	Have you ever hunted flying fox?	Yes, No		Binary
	If yes, what is the reason for hunting?	Food, Traditional medicine, Pest, Income		Categorical
	What is the transport you used to hunt flying fox? (can choose more than one answer)	Boat, Car, Lorry, Motorcycle, Walking		Categorical
	What is the method you used to hunt flying fox? (can choose more than one answer)	Netting, Shooting, Traditional Method, Cut the Tree		Categorical
	When is the time you hunt?	0600 – 0900 hrs, 0901 – 1200 hrs, 1201 – 1500 hrs, 1501 – 1800 hrs, 1801 – 2100 hrs, 2101 – 0000 hrs, 0001 – 0300 hrs, 0301 – 0600 hrs		Categorical
	How many flying fox you ever get in a year?	<10 individuals, 11 – 20 individuals, 21 – 40 individuals, 41 – 60 individuals, 61 – 80 individuals, >80 individuals		Categorical
	How much is a flying fox?	RM10 – 15/individual, RM16 – 30/individual, RM31 – 60/individual, RM61 – 80/individual, RM81 – 100/individual, >RM100/individual		Categorical

Perception	Consuming flying fox meat is good for health.	Strongly disagree, Disagree, Not sure, Agree, Strongly agree		Scale
	Flying fox plays an important role in dispersing seed.	Strongly disagree, Disagree, Not sure, Agree, Strongly agree		Scale
	Flying fox plays an important role in promoting tourism.	Strongly disagree, Disagree, Not sure, Agree, Strongly agree		Scale
	The tourism industry (flying fox) can be enhanced by building jetty in the mangrove forest.	Strongly disagree, Disagree, Not sure, Agree, Strongly agree		Scale
	Tourism activities regard flying fox can increase the local’s economy.	Strongly disagree, Disagree, Not sure, Agree, Strongly agree		Scale
	Occasionally consuming Flying fox meat is fine.	Strongly disagree, Disagree, Not sure, Agree, Strongly agree		Scale
	The hunting and selling of Flying fox can damage Flying fox populations in the long term.	Strongly disagree, Disagree, Not sure, Agree, Strongly agree		Scale
	Deforestation causes a more negative impact on Flying fox population compared to hunting activity.	Strongly disagree, Disagree, Not sure, Agree, Strongly agree		Scale
	Wildlife law is necessary for Sarawak to protect flying fox.	Strongly disagree, Disagree, Not sure, Agree, Strongly agree		Scale
	Besides the Sarawak Wildlife Law, the flying fox also needed to be protected at the village level.	Strongly disagree, Disagree, Not sure, Agree, Strongly agree		Scale
	Awareness program in schools will help to increase the effort to conserve the Flying fox.	Strongly disagree, Disagree, Not sure, Agree, Strongly agree		Scale

### Appendix C

The flying foxes were observed to fly from Brunei (island far back) towards the LMNP.

### Appendix D

The aerial view of LMNP and Kampung Limpaku Pinang.

### Appendix E

The large flying fox (*Pteropus vampyrus*) was photographed at Limbang Mangroves, this tree-roosting species is categorised as “Near Threatened” in IUCN Red List 2020.
